# Decision Tree-Based Foot Orthosis Prescription for Patients with Pes Planus

**DOI:** 10.3390/ijerph191912484

**Published:** 2022-09-30

**Authors:** Ji-Yong Jung, Chang-Min Yang, Jung-Ja Kim

**Affiliations:** 1 Division of Biomedical Engineering, Jeonbuk National University, 567 Baekje-daero, Deokjin-gu, Jeonju-si 54896, Korea; 2 Department of Healthcare Engineering, Jeonbuk National University, 567 Baekje-daero, Deokjin-gu, Jeonju-si 54896, Korea; 3 Research Center of Healthcare & Welfare Instrument for the Aged, Jeonbuk National University, 567 Baekje-daero, Deokjin-gu, Jeonju-si 54896, Korea

**Keywords:** pes planus, foot orthosis, decision tree, classification and regression tree, machine learning

## Abstract

Pes planus, one of the most common foot deformities, includes the loss of the medial arch, misalignment of the rearfoot, and abduction of the forefoot, which negatively affects posture and gait. Foot orthosis, which is effective in normalizing the arch and providing stability during walking, is prescribed for the purpose of treatment and correction. Currently, machine learning technology for classifying and diagnosing foot types is being developed, but it has not yet been applied to the prescription of foot orthosis for the treatment and management of pes planus. Thus, the aim of this study is to propose a model that can prescribe a customized foot orthosis to patients with pes planus by learning from and analyzing various clinical data based on a decision tree algorithm called classification and regressing tree (CART). A total of 8 parameters were selected based on the feature importance, and 15 rules for the prescription of foot orthosis were generated. The proposed model based on the CART algorithm achieved an accuracy of 80.16%. This result suggests that the CART model developed in this study can provide adequate help to clinicians in prescribing foot orthosis easily and accurately for patients with pes planus. In the future, we plan to acquire more clinical data and develop a model that can prescribe more accurate and stable foot orthosis using various machine learning technologies.

## 1. Introduction

With a complex and multifunctional structure, the foot plays an important role in supporting body weight and moving the body very efficiently while standing or walking [1]. Pes planus, commonly known as flat foot, is a structural deformity defined as a condition accompanied by a decrease in the medial longitudinal arch height, rearfoot valgus, and lateral deviation of the forefoot [2]. In previous studies, it has been reported that pes planus is often associated with rotational abnormalities of the lower extremities and increased internal rotation of the hip [3,4]. It is estimated that the prevalence of pes planus is approximately 20% to 37% of the population [5]. This deformation negatively affects the shock absorption from the ground, resulting in foot fatigue, pain, abnormal gait, and postural imbalances [6,7]. In the literature, foot orthosis is most frequently cited as a conservative (non-surgical) intervention for the treatment and management of pes planus [8,9]. A customized foot orthosis has been prescribed based on the diagnosis results of clinicians to control rearfoot alignment, correct posture, provide comfort, and reduce abnormal movement. Bok et al. [10] reported significant improvement in the resting calcaneal stance position (RCSP) of patients with pes planus wearing a customized foot orthosis. Akbari et al. [11] revealed that arch-support orthoses positively affect dynamic postural stability in subjects with flat feet. Xu et al. [12] suggested that wearing a customized orthosis can provide greater comfort than a prefabricated orthosis by effectively distributing foot pressure in the frontal, sagittal, and transverse planes. In addition, Saeedi et al. [13] reported that the use of a modified foot orthosis with a flexible structure for flat-footed patients had a positive effect on the improvement of foot movement in the sagittal and transverse planes.

Recently, with the rapid development of artificial intelligence (AI) technology, its application in the medical field is gradually expanding. Machine learning, a branch of AI technology, uses large amounts of data to learn patterns and predict outcomes. Supervised learning, in which the correct answers are included in the training data, is the most used machine learning algorithm in the medical field due to its high reliability and accuracy [14]. Specifically, it is suitable for classification problems that distinguish between normal and abnormal feet [15]. For this reason, many studies have been conducted on machine learning algorithms that can accurately diagnose foot deformity using various data collected from clinical examination, radiographic evaluation, and footprint analysis. Mei et al. [16] suggested an automatic foot-type classification system, which involves a set of one-dimensional convolutional neural network (CNN) models for extracting discriminative features. It was confirmed that the foot type can be classified with an accuracy of 99.26% based on the combination of sensor measurement data. Eksen et al. [17] proposed a mobile pre-diagnosis system for identifying foot deformities using machine learning-based image recognition algorithms. As a result of classifying the foot type using the prototype based on the automation algorithm, it was found that the accuracy was 91.80%. Chae et al. [15] developed a classification model that utilizes images and numerical data to determine three types of foot deformities.

It was suggested that the combination of two models for image and numerical data analysis in the classification of foot deformities can lead to more accurate diagnostic results than the case of using only a single model. Li et al. [18] presented an improved neural network model with a simple structure to perform pattern classification for flat foot diagnosis. The classification accuracy was 85.29%, which was higher than traditional algorithms such as neural networks and pattern classification. A neural network algorithm was also utilized by Aruntammanak et al. [19] to extract characteristics of the normal and flat feet from a footprint image. This study showed that the classification accuracy can be increased by up to 50% using a combination of indicators related to footprint analysis. Ardhianto et al. [20] implemented a deep learning method using the You Only Look Once (YOLO) algorithm to detect the angle of foot progression angle for evaluating gait abnormalities from plantar pressure images. As such, although machine learning to improve the medical diagnosis accuracy of flat feet is being actively studied, there are still no studies on machine learning for the classification and prescription of a foot orthosis.

A customized foot orthosis is usually prescribed to correct a patient’s rearfoot alignment and movement based on various clinical data (e.g., RCSP, inversion and eversion angles of the subtalar joint, Trendelenburg angle, etc.). Decision tree methods such as classification and regressing tree (CART), Chi-squared automatic interaction detector (CHAID), quick, unbiased, efficient statistical tree (QUEST), commercial version 4.5 (C4.5), and Interactive Dichotomizer version 3 (ID3) are the most effective and practical algorithms for classification and prediction [21]. As a non-parametric technique, the CART model is widely utilized in the medical field. This model is based on the binary split in which the Gini index is calculated, and only two lower nodes are formed from the upper node [22]. In other words, the output value is predicted according to the decision rule for a given input value combination, and it is expressed as a graph of a tree structure. In particular, the CART strategy can be applied to foot orthosis prescription due to its ease of classification by making easy-to-understand rules in an if-then format. Hence, the visualized tree-based model can be effectively utilized for decision-making by physicians about prescribing foot orthoses. Consequently, the purpose of this study was to develop a CART model for foot orthosis prescription with high reliability and accuracy. To the best of our knowledge, this is the first study on the application of a decision tree algorithm to the prescription of a foot orthosis.

## 2. Related Study

Until now, the decision tree-based CART algorithm has been widely used in the medical field. Tange et al. [23] proposed a method for combining CART decision trees with the Boruta algorithm and applying them to medical data classification. In this study, the Boruta algorithm was used to reduce overfitting and error rates that occur when processing datasets with too many categories. Then, the CART algorithm was used to classify the feature subset. From the results, it was confirmed that a combination of algorithms could more accurately classify medical data and help doctors diagnose patients. Xie et al. [24] implemented the CART algorithm to classify the heartbeat of premature ventricular contraction. As a result of classifying diseases using the decision tree, it was found that even with a small number of features, the recognition rate was high, at 99.6%. Aziza et al. [25] developed an automated system based on the CART algorithm to detect diabetic retinopathy after segmenting blood vessels and extracting important geometric features from color fundus images. In conclusion, the diagnostic accuracy of diabetic retinopathy was 93%, which was higher than the existing method, suggesting that it could be helpful in the early diagnosis of the disease. Ghiasi et al. [26] developed a model based on the CART algorithm for coronary artery disease diagnosis. As a result of evaluating the classification performance of the presented CART model, it showed high precision and reliability compared to the existing prediction model. Based on these results, it has been reported that CART classification modeling, which provides easy-to-understand and accurate results, can be usefully used for expert disease diagnosis in actual clinical practice.

## 3. Methodology

### 3.1. CART Algorithm

**Figure 1:**
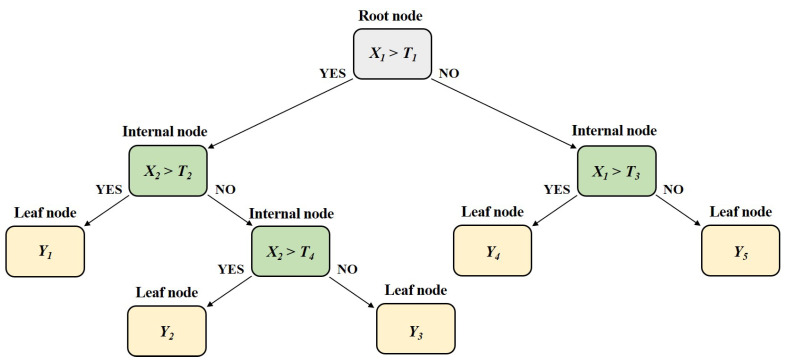
A top-down decision tree.

CART is a representative algorithm for generating decision trees. In practice, various tree-based machine learning methods (Random Forest, Extreme Gradient Boosting, Light Gradient Boosted Machine, Gradient boosting decision tree, etc.) are implemented based on the CART algorithm [27]. This algorithm calculates the Gini index criterion to split a node into a sub-node when constructing a decision tree for classification tasks. The Gini index refers to the probability that two randomly selected variables among *n* input variables belong to different groups. The Gini index (GI) can be expressed as

(1) $$GI=1-\sum_{i=1}^{c}P_{i}^{2}$$

where $P_{i}$ is the probability of class *i*, and *c* is the total class. The CART algorithm is a top-down decision tree, as shown in Figure 1. It is assumed that the independent variables, threshold values, and target variables are indicated by $X_{i}$, $T_{i}$, and $Y_{i}$, respectively. The top node in the tree is called the root node. The internal node, referred to as a decision node, is the point at which the sub-dataset splits into the leaf node. The leaf node is a terminal node that determines the final class (label) value in the tree. A Gini index and weighted sum are calculated for each attribute. Then, the attribute with the lowest Gini index value is selected. As this process repeats, each path from the root node to the leaf node forms a classification rule.

### 3.2. Dataset Description

This study used clinical data from a total of 1548 patients diagnosed with pes planus at the Department of Rehabilitation Medicine of Chungnam National University Hospital in Daejeon, Republic of Korea. Pes planus was diagnosed based on the results measured by a professional clinician with a goniometer (universal goniometer and gravity goniometer) and an inclinometer [28,29,30,31]. Independent variables related to the diagnosis of pes planus in patients include sex, age, hip external rotation (HER), hip internal rotation (HIR), transmalleolar angle on the left side (TMA-L), transmalleolar angle on the left side (TMA-R), inversion angle of the subtalar joint on the left side (IASTJ-L), inversion angle of the subtalar joint on the left side (IASTJ-R), eversion angle of the subtalar joint on the left side (EASTJ-L), eversion angle of the subtalar joint on the right side (EASTJ-R), forefoot to rearfoot angle on the left side (FFRF-L), forefoot to rearfoot angle on the right side (FFRF-R), RCSP angle on the left side (RCSPA-L), RCSP angle on the right side (RCSPA-R), dorsiflexion angle on the left side (DFA-L), dorsiflexion angle on the right side (DFA-R), pelvic elevation angle on the left side (PEA-L), pelvic elevation angle on the right side (PEA-R), pelvic tilting (PT), and pelvic rotation (PR). All variables were preprocessed through importance analysis. Through data preprocessing, the study was conducted with a total of 418 data, excluding data with missing values. Then, 9 variables affecting the prescription of foot orthosis were selected out of 20 independent variables. The selected independent variables include age, HIR, TMA-L, IASTJ-L, EASTJ-L, EASTJ-R, FFRF-R, RCSPA-L, and RCSPA-R.

In this study, two types of foot orthosis (gait plate and arch support orthosis with heel cups) were used as a dependent variable in this study. A gait plate (GP) is a special type of orthosis that is prescribed to limit in-toeing gait due to increased internal hip rotation and femoral anteversion with pes planus [32]. Arch support orthosis with heel cups (ASOHC), designed to support the medial longitudinal arch and heel, is recommended to reduce rearfoot pronation, the collapse of the longitudinal arch, and foot instability [33]. Two types of foot orthoses are customized for each patient’s foot. Plaster casting is one of the techniques for manufacturing foot orthosis that can accurately reflect the shape of a patient’s foot. A plaster cast is made by placing the patient’s foot in a neutral, weight-bearing position and wrapping a wet plaster strip around the foot [6]. Then, based on patient-specific measurements, a GP or ASOHC prescribed by an expert clinician is manufactured.

### 3.3. Dataset Splitting

The dataset was randomly split into two categories: the training dataset (70%) and the testing dataset (30%). A training dataset is the samples required to properly train a CART model. Once a CART classifier is developed, its performance is evaluated prior to application in the clinical field. The testing dataset is used to evaluate the predictive performance of the generated CART model on unobserved data. It is important to note that the model is trained through a 10-fold cross-validation procedure instead of using the validation dataset. Table 1 shows the clinical characteristics of patients with the two types of foot orthosis in the training and test datasets.

### 3.4. Pruning

If the model is overtrained only on the training data, the error in the testing data increases, and its generality decreases. This phenomenon is called overfitting. Pruning is a technique to solve overfitting problems by controlling the complexity of the decision tree. Pruning can be divided into two categories: pre-pruning and post-pruning. Pre-pruning is the process of stopping the growth of a decision tree prematurely to prevent excessive node creation. The pre-pruning method is simple but not practical since it is difficult to accurately determine the end point of tree growth. In the post-pruning approach, a fully grown decision tree is truncated through evaluation criteria and replaced with leaf nodes. The cost complexity pruning (CCP) is the frequently used pruning method in CART. In the pruning process, the prediction accuracy of the decision tree on the training data is calculated, and finally, an optimal tree with a balance between complexity and error rate is obtained. The cost complexity (CC) of a tree *T* is defined as

(2) CC(T)=R(T)+α|T|

where R(T) is the error rate, |T| is the number of leaves on *T*, and the complexity parameter α is the cost of each leaf. The complexity of the tree is adjusted using α. As α increases, the complexity of the tree increases, so many branches are truncated to create a simple model. Conversely, reducing α makes the model slightly more complex.

### 3.5. Evaluation Metrics

The confusion matrix is used to calculate evaluation metrics, including accuracy, sensitivity, precision, and f1 score, and the related equations are [34]:

(3) $$Accuracy=\frac{TP+TN}{TP+TN+FP+FN}$$

gather Sensitivity = TPTP+FN

(4) $$Precision=\frac{TP}{TP+FP}$$

(5) $$F1Score=2*\frac{Sensitivity+Precision}{Sensitivity+Precision}$$

where TP, TN, FP, and FN represent true positive, true negative, false positive, and false negative, respectively. TP is the number of foot orthosis correctly prescribed as GP, TN is the number of foot orthosis incorrectly prescribed as GP, FP is the number of foot orthosis incorrectly prescribed as ASOHC, and FN is the number of foot orthoses correctly prescribed as ASOHC.

**Figure 2:**
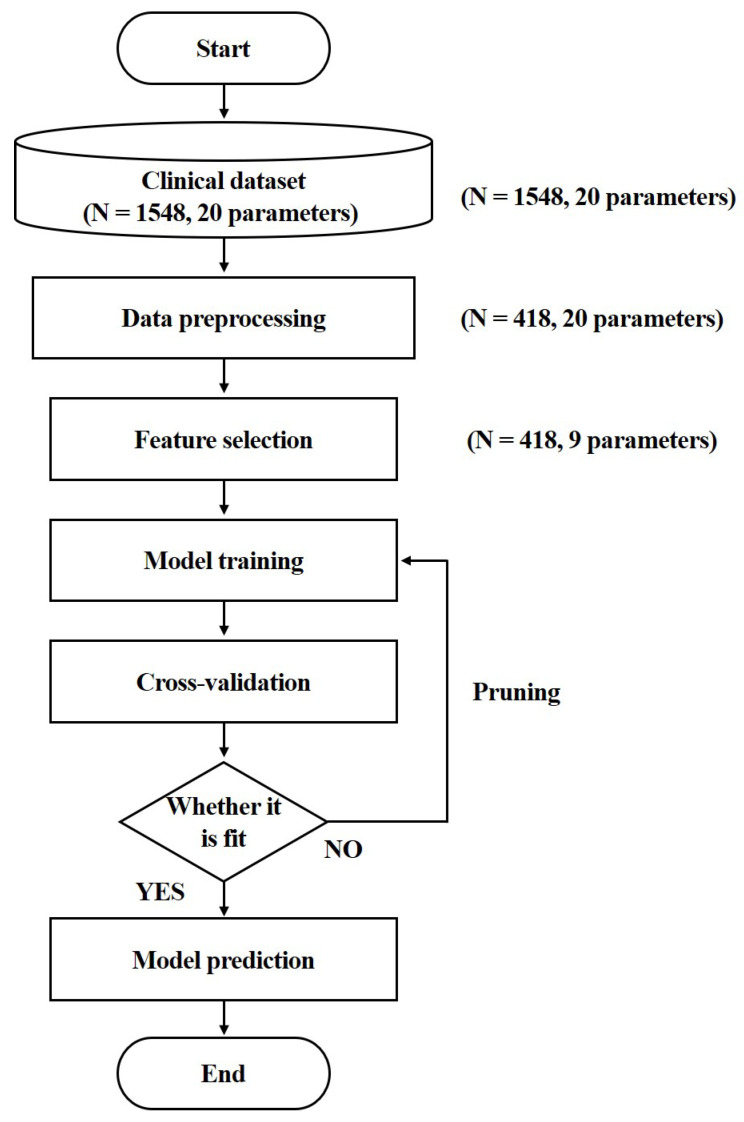
A flow chart of the study procedure.

All the experiments were carried out based on Python 3.10.2 on a 64-bit computer with an Intel i5-7500 (3.4 GHz) CPU and 16 GB RAM. A flow chart of the overall study procedure is shown in Figure 2.

## 4. Results

**Figure 3:**
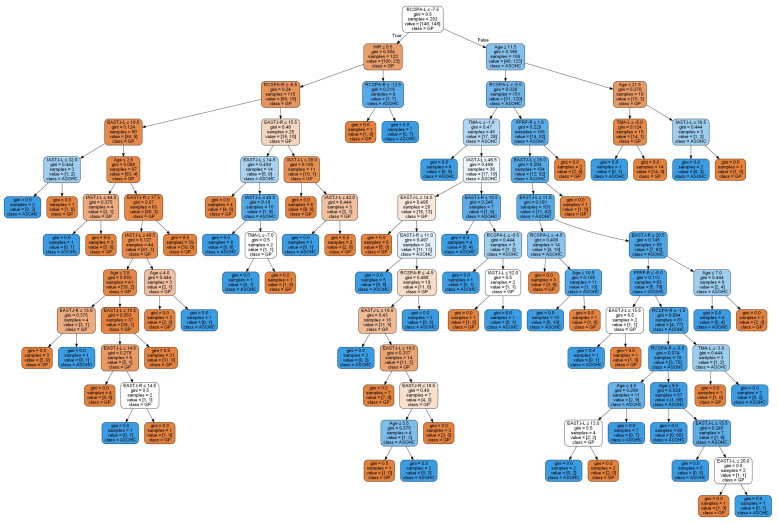
Graphical representation of the CART model before pruning.

**Figure 4:**
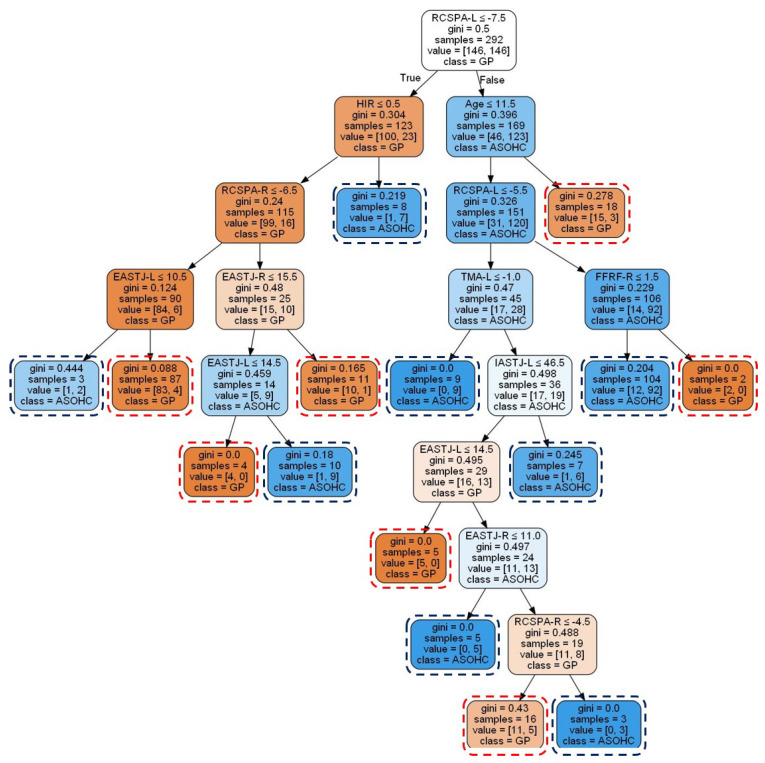
Graphical representation of the CART model after pruning. Note: Gini is a metric that quantifies the purity of the node/leaf. A sample is the number of data. The value represents the number of samples included in the GP and ASOHC at a given node. The color indicates the class to which the majority of samples of each node belong (GP: orange and ASOHC: purple). Darker colors mean lower Gini scores.

Figure 3 shows the graphical representation of the CART model before the pruning of 57 rules that were generated for the prescription of a foot orthosis. On the other hand, as shown in Figure 4, 15 rules were generated for the prescription of foot orthosis based on the CART model after pruning.

The 15 rules for prescribing the two types of foot orthosis (GP or ASOHC) are shown in Table 2. Regarding the GP prescription, 7 rules were created as follows: (1) If `RCSPA-L’ was less than $-7.5{\;}^{\circ }$, `age’ was less than 11.5 years, then `GP’; (2) If `RCSPA-L’ was less than $-7.5{\;}^{\circ }$, `age’ was less than 11.5 years, `RCSPA-L’ was less than $-5.5{\;}^{\circ }$, `FFRF-R’ was less than $1.5{\;}^{\circ }$, then `GP’; (3) If `RCSPA-L’ was less than $-7.5{\;}^{\circ }$, `HIR’ was abnormal, `RCSPA-R’ was less than $-6.5{\;}^{\circ }$, `EASTJ-L’ was less than $10.5{\;}^{\circ }$, then `GP’; (4) If `RCSPA-L’ was less than $-7.5{\;}^{\circ }$, `HIR’ was abnormal, `RCSPA-R’ was less than $-6.5{\;}^{\circ }$, `EASTJ-R’ was less than $15.5{\;}^{\circ }$, then `GP’; (5) If `RCSPA-L’ was less than $-7.5{\;}^{\circ }$, `HIR’ was abnormal, `RCSPA-R’ was less than $-6.5{\;}^{\circ }$, `EASTJ-R’ was less than $15.5{\;}^{\circ }$, `EASTJ-L’ was less than $14.5{\;}^{\circ }$, then `GP’; (6) If `RCSPA-L’ was less than $-7.5{\;}^{\circ }$, age was less than 11.5 years, `RCSPA-L’ was less than $-5.5{\;}^{\circ }$, `TMA-L’ was less than $-1.0{\;}^{\circ }$, `IASTJ-L’ was less than $46.5{\;}^{\circ }$, `EASTJ-L’ was less than $14.5{\;}^{\circ }$, then `GP’; (7) If `RCSPA-L’ was less than $-7.5{\;}^{\circ }$, age was less than 11.5 years, `RCSPA-L’ was less than $-5.5{\;}^{\circ }$, `TMA-L’ was less than $-1.0{\;}^{\circ }$, `IASTJ-L’ was less than $46.5{\;}^{\circ }$, `EASTJ-L’ was less than $14.5{\;}^{\circ }$, `EASTJ-R’ was less than $11.0{\;}^{\circ }$, `RCSPA-R’ was less than $-4.5{\;}^{\circ }$, then `GP’. In addition, with regard to the ASOHC prescription, 8 rules were created as follows: (1) If `RCSPA-L’ was less than $-7.5{\;}^{\circ }$, `HIR’ was abnormal, then `ASOHC’; (2) If `RCSPA-L’ was less than $-7.5{\;}^{\circ }$, `age’ was less than 11.5 years, `RCSPA-L’ was less than $-5.5{\;}^{\circ }$, `TMA-L’ was less than $-1.0{\;}^{\circ }$, then `ASOHC’; (3) If `RCSPA-L’ was less than $-7.5{\;}^{\circ }$, `age’ was less than 11.5 years, `RCSPA-L’ was less than $-5.5{\;}^{\circ }$, `FFRF-R’ was less than $1.5{\;}^{\circ }$, then `ASOHC’; (4) If `RCSPA-L’ was less than $-7.5{\;}^{\circ }$, `HIR’ was abnormal, `RCSPA-R’ was less than $-6.5{\;}^{\circ }$, `EASTJ-L’ was less than $10.5{\;}^{\circ }$, then `ASOHC’; (5) If `RCSPA-L’ was less than $-7.5{\;}^{\circ }$, `HIR’ was abnormal, `RCSPA-R’ was less than $-6.5{\;}^{\circ }$, `EASTJ-R’ was less than $15.5{\;}^{\circ }$, `EASTJ-L’ was less than $14.5{\;}^{\circ }$, then `ASOHC’; (6) If `RCSPA-L’ was less than $-7.5{\;}^{\circ }$, `age’ was less than 11.5 years, `RCSPA-L’ was less than $-5.5{\;}^{\circ }$, `TMA-L’ was less than $-1.0{\;}^{\circ }$, `IASTJ-L’ was less than $46.5{\;}^{\circ }$, then `ASOHC’; (7) If `RCSPA-L’ was less than $-7.5{\;}^{\circ }$, `age’ was less than 11.5 years, `RCSPA-L’ was less than $-5.5{\;}^{\circ }$, `TMA-L’ was less than $-1.0{\;}^{\circ }$, `IASTJ-L’ was less than $46.5{\;}^{\circ }$, `EASTJ-L’ was less than $14.5{\;}^{\circ }$, `EASTJ-R’ was less than $11.0{\;}^{\circ }$, then `ASOHC’; (8) If `RCSPA-L’ was less than $-7.5{\;}^{\circ }$, `age’ was less than 11.5 years, `RCSPA-L’ was less than $-5.5{\;}^{\circ }$, `TMA-L’ was less than $-1.0{\;}^{\circ }$, `IASTJ-L’ was less than $46.5{\;}^{\circ }$, `EASTJ-L’ was less than $14.5{\;}^{\circ }$, `EASTJ-R’ was less than $11.0{\;}^{\circ }$, `RSCPA-R’ was less than $-4.5{\;}^{\circ }$, then ’ASOHC’.

Table 3 shows the performance of the proposed CART model for foot orthosis prescription. The accuracy of the foot orthosis prescription for patients with pes planus, based on the CART algorithm, is 80.16%. The results of GP prescription showed 89.66% precision, 73.24% sensitivity, and 80.62% f1 score. In the results of ASOHC prescription, the precision, sensitivity, and f1 score, were 72.06%, 89.09%, and 79.67%, respectively.

**Figure 5:**
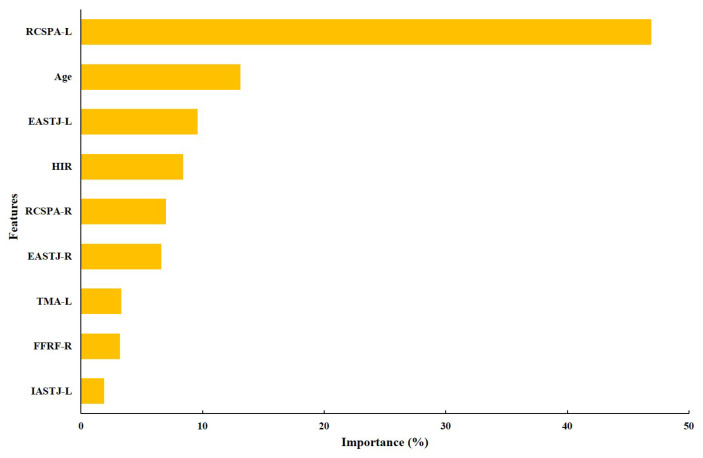
Feature importance.

Figure 5 presents the importance of each feature of the CART model. The importance ratio of RCSPA-L was the highest at 46.9%. The importance ratio for other features showed 13.1% for age, 9.6% for EASTJ-L, 8.4% for HIR, 7% for RCSPA-R, 6.6% for EASTJ-R, 3.3% for TMA-L, 3.2% for FFRF-R, and 1.9% for IASTJ-L.

## 5. Discussion

In this study, we confirmed the classification accuracy of the CART model for foot orthosis prescription using evaluation metrics. As a result, it was found that the classification accuracy was relatively low compared to the previous study. Previous studies have mainly proposed a method for learning and classifying foot types using sensors or image data. Although these data can provide accurate information for determining whether a patient has pes planus, there are limitations in understanding the physical characteristics or functional movements of a patient with pes planus [35]. In this study, clinical characteristics (age, angle, range of motion) of patients with pes planus were measured by an expert clinician and used as data. Therefore, it is very useful because it can be utilized not only for the diagnosis of pes planus but also for the prescription of foot orthoses. In addition, it has been suggested that the appropriate treatment and management for patients with pes planus can be very complex, as the etiology itself is not clear [36]. Considering this point, it is believed that detailed factors such as the types of pes planus (rigid or flexible), period (congenital or acquired), and comorbidities (scoliosis, pelvic asymmetry, leg length discrepancy, etc.) were not considered in this study may affect the accuracy of prescription.

We developed the CART model that prescribes two types of foot orthoses in consideration of the various biomechanical characteristics of patients with pes planus. Based on the evaluation results on feature importance, it was confirmed that RCSPA, age, EASTJ, HIR, TMA, FFRF, and IASTJ were important parameters in prescribing foot orthosis. The relatively high importance of the RCSP angle may be due to the prescription of the footrest to increase the arch height and adjust the alignment of the rearfoot [10,37]. Age has also been found to be an important factor in prescribing foot orthosis. This result is consistent with previous studies suggesting that excessive joint mobility affecting arch height reduction, rearfoot valgus, and forefoot abduction can manifest differently with age [38]. When comparing the clinical characteristics of the patients according to the type of foot orthosis, the RCSPA and EASTJ values of the patients who were prescribed ASOHC were higher. Previous studies have shown that arch support and heel cup are effective in improving the collapse of the longitudinal arch and excessive pronation due to increased eversion angle of the subtalar joint and calcaneus [33]. On the other hand, the TMA values were higher in the patients who were prescribed GP. Pes planus has been associated with rotational abnormalities of the lower extremities (femoral anteversion and tibial torsion), which are common causes of intoeing gait [39]. In previous studies, it was confirmed that GP is a very effective foot orthosis for improving gait appearance in patients with an abnormal gait pattern [32,40]. As mentioned above, although detailed factors for a patient may affect the accuracy of prescription, the CART model for prescribing a foot orthosis in consideration of the importance and measurement value for biomechanical variables can be utilized to assist expert clinicians.

## 6. Limitations

The limitations of this study are as follows. First, there were insufficient quality training data for patients with pes planus. Second, the types of pes planus (congenital or acquired/rigid or flexible), the time of onset, and other diseases related to pes planus were not considered. Finally, there are no indicators to evaluate the clinical effectiveness of a prescribed foot orthosis. Therefore, in future research, based on high-quality training data, we will develop a machine learning algorithm that can compare and analyze the difference in foot shape and functional movement of patients with pes planus before and after the prescription of a foot orthosis.

## 7. Conclusions

As interest in healthcare has recently increased, the need for technology that can provide disease prediction, preventive medicine, and patient-specific treatment based on learning and the analysis of vast amounts of data is being emphasized. CART is one of the most-used algorithms in the medical field because of its excellent performance in analyzing training data and predicting and classifying new data with unique patterns in the data. In this study, we present a CART algorithm-based method that can prescribe a customized foot orthosis to flat-footed patients with relatively high prevalence. As a result, 15 rules were generated based on the importance of the 9 variables related to foot orthosis prescription identified. The main advantage of the CART algorithm-based foot orthosis prescription strategy proposed in this study is that the visualized results can be helpful in the decision-making process of experts. The results of this study support that the CART model, which can be easily and quickly understood, can be effectively utilized when a clinician prescribes a foot orthosis. It is expected that more accurate and stable foot orthosis prescriptions will be possible if more clinical data are secured, and a new model incorporating various machine learning technologies is presented in the future.

## Figures and Tables

**Table 1:** Clinical characteristics of patients with the two types of foot orthoses in the training and test datasets.

**Characteristics**		**Training Dataset (** ***n*** ** = 292)**		**Test Dataset (** ***n*** ** = 126)**	
**Name**	**Description**	**GP (** ***n*** ** = 146)**	**ASOHC (** ***n*** ** = 146)**	**GP (** ***n*** ** = 71)**	**ASOHC (** ***n*** ** = 55)**
Age (year)	Age	6.52 ± 4.01	8.24 ± 3.85	6.38 ± 3.31	7.41 ± 4.37
HIR (0 or 1)	Hip internal rotation angle (>$45{\;}^{\circ }$: abnormal (0), ≤$45{\;}^{\circ }$: normal (1))	0 (*n* = 110) 1 (*n* = 36)	0 (*n* = 144) 1 (*n* = 2)	0 (*n* = 55) 1 (*n* = 16)	0 (*n* = 52) 1 (*n* = 3)
TMA-L (degree)	Transmalleolar angle on the left side	−1.93 ± 5.46	−0.78 ± 2.92	−2.03 ± 5.12	−0.22 ± 2.23
IASTJ-L (degree)	Inversion angle of the subtalar joint on the left side	37.82 ± 7.88	38.64 ± 7.02	38.09 ± 7.62	38.45 ± 8.10
EASTJ-L (degree)	Eversion angle of the subtalar joint on the left side	16.21 ± 3.91	18.29 ± 4.26	15.63 ± 3.92	17.45 ± 3.51
EASTJ-R (degree)	Eversion angle of the subtalar joint on the right side	14.49 ± 4.21	16.86 ± 4.42	14.82 ± 4.55	15.95 ± 4.52
FFRF-R (degree)	forefoot to rearfoot angle on the right side	0.02 ± 0.26	0.12 ± 0.58	0.07 ± 0.59	0.27 ± 0.89
RCSPA-L (degree)	RCSP angle on the left side	−5.08 ± 2.45	−9.19 ± 3.75	−5.29 ± 2.95	−9.30 ± 3.33
RCSPA-R (degree)	RCSP angle on the right side	−4.58 ± 2.00	−8.12 ± 3.39	−4.62 ± 2.51	−8.18 ± 3.38

**Table 2:** The 15 rules for the prescription of foot orthoses.

Rules			
GP		1	RCSPA-L ≤ $-7.5{\;}^{\circ }$, Age ≤ 12 years
		2	RCSPA-L ≤ $-7.5{\;}^{\circ }$, Age ≤ 12 years, RCSPA-L ≤ $-5.5{\;}^{\circ }$, FFRF-R ≤ $1.5{\;}^{\circ }$
		3	RCSPA-L ≤ $-7.5{\;}^{\circ }$, HIR = abnormal, RCSPA-R ≤ $-6.5{\;}^{\circ }$, EASTJ-L ≤ $10.5{\;}^{\circ }$
		4	RCSPA-L ≤ $-7.5{\;}^{\circ }$, HIR = abnormal, RCSPA-R ≤ $-6.5{\;}^{\circ }$, EASTJ-R ≤ $15.5{\;}^{\circ }$
		5	RCSPA-L ≤ $-7.5{\;}^{\circ }$, HIR = abnormal, RSCPA-R ≤ $-6.5{\;}^{\circ }$, EASTJ-R ≤ $15.5{\;}^{\circ }$, EASTJ-L ≤ $14.5{\;}^{\circ }$
		6	RCSPA-L ≤ $-7.5{\;}^{\circ }$, Age ≤ 12 years, RCSPA-L ≤ $-5.5{\;}^{\circ }$, TMA-L ≤ $-1.0{\;}^{\circ }$, IASTJ-L ≤ $46.5{\;}^{\circ }$, EASTJ-L ≤ $14.5{\;}^{\circ }$
		7	RCSPA-L ≤ $-7.5{\;}^{\circ }$, Age ≤ 12 years, RCSPA-L ≤ $-5.5{\;}^{\circ }$, TMA-L ≤ $-1.0{\;}^{\circ }$, IASTJ-L ≤ $46.5{\;}^{\circ }$, EASTJ-L ≤ $14.5{\;}^{\circ }$, EASTJ-R ≤ $11.0{\;}^{\circ }$, RCSPA-R ≤ $-4.5{\;}^{\circ }$
ASOHC		1	RCSPA-L ≤ $-7.5{\;}^{\circ }$, HIR = Normal
		2	RCSPA-L ≤ $-7.5{\;}^{\circ }$, Age ≤ 12 years, RCSPA-L ≤ $-5.5{\;}^{\circ }$, TMA-L ≤ $-1.0{\;}^{\circ }$
		3	RCSPA-L ≤ $-7.5{\;}^{\circ }$, Age ≤ 12 years, RCSPA-L ≤ $-5.5{\;}^{\circ }$, FFRF-R ≤ $1.5{\;}^{\circ }$
		4	RCSPA-L ≤ $-7.5{\;}^{\circ }$, HIR = abnormal, RCSPA-R ≤ $-6.5{\;}^{\circ }$, EASTJ-L ≤ $10.5{\;}^{\circ }$
		5	RCSPA-L ≤ $-7.5{\;}^{\circ }$, HIR = abnormal, RCSPA-R ≤ $-6.5{\;}^{\circ }$, EASTJ-R ≤ $15.5{\;}^{\circ }$, EASTJ-L ≤ $14.5{\;}^{\circ }$
		6	RCSPA-L ≤ $-7.5{\;}^{\circ }$, Age ≤ 12 years, RCSPA-L ≤ $-5.5{\;}^{\circ }$, TMA-L ≤ $-1.0{\;}^{\circ }$, IASTJ-L ≤ $46.5{\;}^{\circ }$
		7	RCSPA-L ≤ $-7.5{\;}^{\circ }$, Age ≤ 12 years, RCSPA-L ≤ $-5.5{\;}^{\circ }$, TMA-L ≤ $-1.0{\;}^{\circ }$, IASTJ-L ≤ $46.5{\;}^{\circ }$, EASTJ-L ≤ $14.5{\;}^{\circ }$, EASTJ-R ≤ $11.0{\;}^{\circ }$
		8	RCSPA-L ≤ $-7.5{\;}^{\circ }$, Age ≤ 12 years, RCSPA-L ≤ $-5.5{\;}^{\circ }$, TMA-L ≤ $-1.0{\;}^{\circ }$, IASTJ-L ≤ $46.5{\;}^{\circ }$, EASTJ-L ≤ $14.5{\;}^{\circ }$, EASTJ-R ≤ $11.0{\;}^{\circ }$, RCSPA-R ≤ $-4.5{\;}^{\circ }$

**Table 3:** Evaluation metrics for classification using the CART model.

Class	Accuracy (%)	Precision (%)	Sensitivity (%)	F1 Score (%)
GP	80.16	89.66	73.24	80.62
ASOHC	80.16	72.06	89.09	79.67

## Data Availability

The data in this study are available on request from the corresponding author.
